# Cell Density-Dependent Suppression of Perlecan and Biglycan Expression by Gold Nanocluster in Vascular Endothelial Cells

**DOI:** 10.3390/cells15020209

**Published:** 2026-01-22

**Authors:** Takato Hara, Misato Saeki, Misaki Shirai, Yuichi Negishi, Chika Yamamoto, Toshiyuki Kaji

**Affiliations:** 1 Faculty of Pharmaceutical Sciences, Toho University, 2-2-1 Miyama, Funabashi 274-8510, Japan; takato.hara@phar.toho-u.ac.jp (T.H.); 3023004s@st.toho-u.ac.jp (M.S.); 2 Faculty of Pharmaceutical Sciences, Tokyo University of Science, 6-3-1 Niijuku, Katsushika-ku, Tokyo 125-8585, Japan; 3 Japanese Society for the Promotion of Science, 5-3-1 Kojimachi, Chiyoda-ku, Tokyo 102-0083, Japan; 4 Institute of Multidisciplinary Research for Advanced Materials, Tohoku University, 2-1-1 Katahira, Aoba-ku, Sendai 980-8577, Japan; yuichi.negishi.a8@tohoku.ac.jp

**Keywords:** gold-nanocluster, proteoglycan, Arf6, vascular endothelial cell

## Abstract

**Highlights:**

**What are the main findings?**

- Au_25_(SG)_18_, a nanoscale gold cluster with low electrophilicity, accumulates in vascular endothelial cells at low cell density and suppresses perlecan expression by inactivating ADP-ribosylation factor 6 (Arf6).
- Au_25_(SG)_18_ also decreases biglycan expression in vascular endothelial cells at low cell density; however, the underlying mechanisms remain unclear.

**What are the implications of the main findings?**

- This study suggests that organic–inorganic hybrid molecules modulate Arf6-mediated protein transport to the extracellular space, revealing a novel mechanism for proteoglycan synthesis regulation.
- Arf6-mediated extracellular transport plays a critical role in maintaining vascular homeostasis, serving as a potential target to modulate endothelial function and vascular diseases.

**Abstract:**

Proteoglycans are macromolecules consisting of a core protein and one or more glycosaminoglycan side chains. Proteoglycans synthesized by vascular endothelial cells modulate various functions such as anticoagulant activity and vascular permeability. We previously reported that some heavy metals interfere with proteoglycan expression, and that organic–inorganic hybrid molecules, such as metal complexes and organometallic compounds, serve as useful tools to analyze proteoglycan synthesis mechanisms. However, the effects of metal compounds lacking electrophilicity on proteoglycan synthesis remain unclear. Au_25_(SG)_18_, a nanoscale gold cluster consisting of a metal core protected by gold–glutathione complexes, exhibits extremely low intramolecular polarity. In this study, we investigated the effect of Au_25_(SG)_18_ on proteoglycan synthesis in vascular endothelial cells. Au_25_(SG)_18_ accumulated significantly in vascular endothelial cells at low cell density and suppressed the expression of perlecan, a major heparan sulfate proteoglycan in cells, by inactivating ADP-ribosylation factor 6 (Arf6). Additionally, Au_25_(SG)_18_ reduced the expression of biglycan, a small dermatan sulfate proteoglycan, in vascular endothelial cells at low cell density; however, the underlying mechanisms remain unclear. Overall, our findings suggest that organic–inorganic hybrid molecules regulate the activity of Arf6-mediated protein transport to the extracellular space and that perlecan is regulated through this mechanism, highlighting the importance of Arf6-mediated extracellular transport for maintaining vascular homeostasis.

## 1. Introduction

Vascular endothelial cells covering the lumen of blood vessels in a monolayer function as a barrier between the blood and subendothelial matrix to allow specific plasma components to penetrate tissues [1]. The endothelial monolayer also regulates the blood coagulation–fibrinolytic system by secreting the procoagulant tissue factor [2], prostacyclin [3], which inhibits platelet aggregation, anticoagulant thrombomodulin [4], and fibrinolytic tissue plasminogen activator [5] and its inhibitor (plasminogen activator inhibitor-1) [6]. Proteoglycans (PGs) are macromolecules consisting of glycosaminoglycan (GAG) chains that are covalently bound to a core protein backbone. They are ubiquitously present in the extracellular matrix and cell membranes of animal tissues [7]. These PG molecules are classified based on the type of GAG chain attached to their core protein. Vascular endothelial cells predominantly synthesize two types of PGs: heparan sulfate PGs (HSPGs) and chondroitin/dermatan sulfate PGs (CS/DSPGs) [8]. These HSPGs include perlecan, a large molecular species constituting the basement membrane [9], and the transmembrane syndecan-1 and syndecan-4 [10]. Biglycan is the primary DSPG secreted into the extracellular space [11]. Vascular endothelial cells predominantly synthesize perlecan and biglycan [8]. Another DSPG, decorin [12] and a large CSPG, versican [13], are expressed under specific conditions such as angiogenesis and hypoxia, respectively. Endothelial PGs contribute to the anticoagulant properties of the vascular endothelium [14,15]. Specifically, heparan sulfate chains of perlecan and syndecan family bind to and activate antithrombin III to inhibit thrombin, which converts fibrinogen into fibrin [16]. Meanwhile, dermatan sulfate chains of biglycan activate heparin cofactor II, thereby inhibiting thrombin [17]. Considering the important role of PGs in maintaining the anticoagulant properties and tone of blood vessels [18], their synthesis mechanisms in cells warrant further investigation.

Endothelial PG synthesis is regulated by various growth factors/cytokines, including transforming growth factor (TGF)-β_1_ [19,20], fibroblast growth factor (FGF)-2 [21], connective tissue growth factor [22], vascular endothelial growth factor-165 [23], tumor necrosis factor-α [24], and interleukin-1β [25]. Each growth factor/cytokine regulates the synthesis of specific PG types, depending on the cell density. For example, TGF-β_1_ induces the synthesis of both perlecan and biglycan at high cell density and only biglycan at low cell density. Vascular endothelial growth factor-165 selectively induces perlecan synthesis [23], connective tissue growth factor suppresses biglycan synthesis but induces decorin synthesis in cells at low cell densities [22], and FGF-2 induces syndecan-4 synthesis via different molecular mechanisms depending on the cell density [26].

In addition to growth factors and cytokines, some heavy metals also alter the synthesis of endothelial PG types. Perlecan synthesis was found to be induced by cadmium [27]. In contrast, lead suppressed perlecan synthesis in vascular endothelial cells [28]. This suppression by lead was mediated by the epidermal growth factor receptor–extracellular signal-regulated kinase (ERK)-1/2–cyclooxygenase-2–prostaglandin I_2_ pathway [29] and inhibited the proliferation of vascular endothelial cells by decreasing the availability of endogenous FGF-2 [30]. These results suggest that some cationic heavy metals influence endothelial PG synthesis. Therefore, we investigated whether organic–inorganic hybrid molecules affect PG synthesis in endothelial cells. Copper (II) bis(diethyldithiocarbamate) induced syndecan-4 synthesis via activation of p38 mitogen-activated protein kinase (MAPK) in vascular endothelial cells [31]. Syndecan-4 synthesis was also induced by organic–inorganic hybrid molecules with a 1,10-phenanthroline structure activation of the hypoxia-inducible factor-1α/β pathway via inhibition of prolyl hydroxylase-domain-containing protein 2 [32]. Dichloro(2,9-dimethyl-1,10-phenanthroline)zinc(II) modulates endothelial perlecan synthesis depending on the cell density [33]. *Sb*-Phenyl-*N*-methyl-5,6,7,12-tetrahydrodibenz[*c,f*][1,5]azastibocine also induced perlecan synthesis in vascular endothelial cells at high cell density [34]. All inorganic metals and organometallic compounds that we have identified to date possess electrophilic properties. However, whether the expression of PG molecular species is regulated by nonelectrophilic compounds remains unclear.

Recently, nanotechnology has made remarkable progress, showing potential to solve societal issues in various fields, such as materials, energy, environment, information communication, and healthcare [35]. Consequently, the development of stable and highly functional metal nanoclusters consisting of several hundred metal atoms within 1–2 nm has also made considerable progress. Particularly, gold nanoclusters exhibit high-functioning properties unique to their size that are not observed in metals other than gold, such as catalytic activity [36,37], optical activity [38], luminescent properties [39,40], and magnetic properties [41,42]. These nanomaterials have attracted significant attention in various fields such as fuel cells, photocatalysts, and solar cells. Reports on the biological activity of gold nanoparticles (AuNPs) exist [43]; however, little is known about the biological activity of gold nanoclusters. Thiolate-protected gold clusters (Au_n_(SR)_m_) exhibit a structure in which a gold–thiolate complex (-SR-Au-SR-) is bound to the surface of the gold core, and Au-Au and Au-SR bonds form a rigid cyclic network, providing high stability [44]. Particularly stable clusters composed of gold atoms with magic numbers (*n* = 25, 38, 102, and 144) have been identified [45,46]. Glutathione-protected Au_25_(SG)_18_ exhibits a diameter of approximately 1 nm [47], and its gold core, which consists of 13 gold atoms in an icosahedral structure, exhibits minimal polarity within the molecule [48]. These reports suggest that Au_25_(SG)_18_ is a non-electrophilic metal compound. In this study, we aimed to determine the effect of this gold cluster Au_25_(SG)_18_ on PG synthesis in vascular endothelial cells.

## 2. Materials and Methods

### 2.1. Materials

Bovine aortic endothelial cells were obtained from Cell Applications (San Diego, CA, USA). Tissue culture dishes and plates were purchased from AGC Techno Glass (Shizuoka, Japan). Dulbecco’s modified Eagle’s medium (DMEM) and Ca^2+^- and Mg^2+^-free phosphate-buffered saline were obtained from Nissui Pharmaceutical (Tokyo, Japan). Au_25_(SG)_18_ was synthesized as previously described [49]. Standard gold nanoparticles (5 nm) were purchased from Cytodiagnostics (Burlington, ON, Canada). Universal negative control small interfering RNA (siRNA), GeneAce SYBR qPCR Mix α, and ISOGEN II were purchased from NipponGene (Tokyo, Japan). Sulfur-35 ([^35^S]Na_2_SO_4_; NEX041H) and Insta-Gel Plus were obtained from PerkinElmer (Waltham, MA, USA). GGA3-PBD Agarose Beads were purchased from Cell Biolabs (San Diego, CA, USA), and Chondroitinase ABC was obtained from Sigma-Aldrich (St. Louis, MO, USA). Fetal bovine serum, Lipofectamine RNAiMAX, high-capacity cDNA reverse transcription kit, MagicMark XP Western Protein Standard, and bicinchoninic acid (BCA) protein assay kit were purchased from Thermo Fisher Scientific (Waltham, MA, USA). Amersham Hybond P 0.2-µm polyvinylidene difluoride and diethylaminoethyl-Sephacel were obtained from GE Healthcare (Chicago, IL, USA). Heparinase II and III were purchased from IBEX Pharmaceuticals (Quebec, QC, Canada). Anti-biglycan (ab94460) and anti-Arf6 (EPR8357; ab131261) antibodies were obtained from Abcam (Cambridge, UK). Anti-glyceraldehyde-3-phosphate dehydrogenase antibody was purchased from Wako Pure Chemical Industries (Osaka, Japan). Anti-perlecan antibody (E-6; sc-377219) was obtained from Santa Cruz Biotechnology (Santa Cruz, CA, USA). Anti-rabbit (#7074) and anti-mouse (#7076) secondary antibodies were purchased from Cell Signaling Technology (Danvers, MA, USA). Chemi-Lumi One Super and other reagents of the highest grade available were obtained from Nacalai Tesque (Kyoto, Japan).

### 2.2. Cell Culture and siRNA Transfection

Vascular endothelial cells were cultured in DMEM supplemented with 10% fetal bovine serum at 37 °C in a humidified atmosphere of 5% CO_2_ until confluence. Subsequently, the cells were transferred to 100 mm dishes and 24-well plates at a density of 1 × 10^4^ cells/cm^2^ and cultured for 24 h (sparse culture) or 35 and 60 mm dishes and 24-well plates until confluence (dense culture), at an approximate density of 1 × 10^5^ cells/cm^2^. In another experiment, the cells were cultured in DMEM supplemented with 10% fetal bovine serum at 37 °C in a humidified atmosphere of 5% CO_2_ until 80–90% confluence. Next, siRNA was transfected into the cells using Lipofectamine RNAiMAX, as previously described [20]. The final concentrations of siRNA and Lipofectamine RNAiMAX were 30 nM and 0.15%, respectively. The following sequences of siRNA sense and antisense strands were used: Bovine *Arf6* siRNA, 5′-CGGTCATTGATAATGCGGdTdT-3′ (sense) and 5′-GCACCGCAUUAUCAAUGACdTdT-3′ (antisense). After 24 h of incubation, the transfected cells were transferred to 60 mm dishes at densities of 1 and 6 × 10^4^ cells/cm^2^, cultured for 24 h, and used for subsequent experiments.

### 2.3. Incorporation of [^35^S]Sulfate into GAGs

Dense and sparse cultures of vascular endothelial cells were seeded in 24-well plates. After washing twice with serum-free DMEM, the cells were treated with various concentrations of Au_25_(SG)_18_ (0.05, 0.10, 0.20, 0.39, and 0.78 µg/mL) in fresh serum-free DMEM and incubated in the presence of [^35^S]sulfate (1 MBq/mL) for 24 h. Subsequently, [^35^S]sulfate incorporation into GAGs was determined via cetylpyridinium chloride precipitation, as previously described [19].

### 2.4. Quantitative Reverse Transcription-Polymerase Chain Reaction (qRT-PCR)

Dense and sparse cultures of vascular endothelial cells cultured in 35 and 100 mm dishes, respectively, were treated with various concentrations of Au_25_(SG)_18_ (0.05, 0.1, 0.2, 0.39, 0.78, 3.13, and 12.5 µg/mL) in fresh serum-free DMEM for 2, 4, 8, 12, and 24 h. Then, total RNA was extracted from the cells and reverse-transcribed into cDNA as previously described [50]. For this analysis, qRT-PCR was performed on the CFX Connect real-time PCR system (BioRad; Hercules, CA, USA) using the GeneAce SYBR qPCR mix α with 5 ng of cDNA and 0.2 µM of the following primers: Bovine perlecan, 5′-ATGGCAGCGATGAAGCGGAC-3′ (forward) and 5′-TTGTGGACACGCAGCGGAAC-3′ (reverse); bovine biglycan, 5′-GCTGCCACTGCCATCTGAG-3′ (forward) and 5′-CGAGGACCAAGGCGTAG-3′ (reverse); bovine syndecan-1, 5′-GGGGACGACAGTGACAACTTCTC-3′ (forward) and 5′-CCTCCTTCTGGGCGGTGA-3′ (reverse); bovine syndecan-4, 5′-TTGCCGTCTTCCTCGTGC-3′ (forward) and 5′-AGGCGTAGAACTCATTGGTGG-3′ (reverse); bovine beta-2-microglobulin (*B2M*), 5′-CCATCCAGCGTCCTCCAAAGA-3′ (forward) and 5′-TTCAATCTGGGGTGGATGGAA-3′ (reverse). The mRNA levels of perlecan, biglycan, syndecan-1, syndecan-4, and *B2M* were quantified using the relative standard curve method. The fold change in target gene intensity was normalized to *B2M* intensity.

### 2.5. PG Core Protein Expression and Western Blotting Analysis

Sparse vascular endothelial cells were prepared in 100 mm dishes. The cells were washed twice with serum-free DMEM and treated with 0.20 and 0.78 µg/mL Au_25_(SG)_18_ in fresh serum-free DMEM for 24 h. After treatment, PGs that accumulated in the cell layer were extracted from the whole-cell lysate, and the supernatant after culture (conditioned medium) of vascular endothelial cells was concentrated as previously described [20]. Concentrated PGs were digested with heparinase II/III in 100 mM Tris-HCl (pH 7.0) containing 10 mM calcium acetate and 18 mM sodium acetate (for perlecan core protein) or chondroitinase ABC in 50 mM Tris-HCl (pH 8.0) containing 1 mg/mL bovine serum albumin and 3 mM sodium acetate (for perlecan core protein). After digesting the heparan and chondroitin/dermatan sulfate chains with heparinase II/III and chondroitinase ABC, respectively, PG core protein bands were detected via western blotting analysis, and the molecular mass of the PG core protein was determined via sodium dodecyl sulfate-polyacrylamide gel electrophoresis. An overview of the experimental workflow is shown in Figure S1A. Western blotting analysis was performed on samples obtained from multiple independent experiments at least twice to confirm reproducibility.

### 2.6. Intracellular Accumulation of Gold Atoms

Vascular endothelial cells transfected siRNA were treated with 0.78 µg/mL of Au_25_(SG)_18_ in fresh serum-free DMEM. After a 24-h incubation, the cell layer was harvested with 80 µL of 50 mM Tris-HCl buffer solution (pH 6.8) containing 2% sodium dodecyl sulfate and 10% glycerol. The samples were incubated as previously described [51] and dissolved in 5 mL of 0.1 M HNO_3_. The gold atom content was analyzed by Nexion 300S inductively coupled plasma mass spectrometry (PerkinElmer; Waltham, MA, USA) under conditions optimized for a plasma output of 1600 W, plasma gas flow of 18.0 L/min, and nebulizer gas flow rate of 0.94 L/min. Another portion of the cell lysate was analyzed for protein content using the BCA protein assay kit to express gold content as pmol/mg protein.

### 2.7. Arf6 Activation (GTP-Bound Arf6 Pulldown) Assay

Sparse cultures of vascular endothelial cells grown in 100 mm dishes were treated with 0.2 and 0.78 µg/mL Au_25_(SG)_18_ for 12 h in fresh serum-free DMEM. After incubation, the cells were washed twice with Ca^2+^- and Mg^2+^-free phosphate-buffered saline and lysed with 100 µL of assay/lysis buffer (25 mM HEPES [pH 7.5], 150 mM NaCl, 10 mM MgCl_2_, 1 mM EDTA, 2% glycerol, and 1% NP-40). The lysates were centrifuged at 14,000× *g* for 10 min at 4 °C. Protein concentrations in the collected supernatants were determined using the BCA protein assay kit. The samples were diluted to 400 µg protein in 1 mL with the assay/lysis buffer. The negative control sample was prepared according to the protocol of the Arf6 Activation Assay Kit (STA-407-6; Cell Biolabs). To each sample, 40 µL of GGA3-PBD Agarose Bead solution was added and rotated at 4°C for 1 h. Then, the samples were centrifuged at 14,000× *g* for 3 min at 4 °C, and the supernatants were discarded. The precipitated beads were washed with 500 µL of the assay/lysis buffer. The washing step was repeated thrice. After the final wash, 20 µL of loading buffer (50 mM Tris [pH 6.8], 8% glycerol, 2% sodium dodecyl sulfate, 5% 2-mercaptoethanol, and 0.002% bromophenol blue) were added to each sample. The samples were boiled at 95°C for 5 min. Finally, GTP-bound Arf6 protein expression was measured by western blotting analysis as described above. An overview of the experimental workflow is shown in Figure S1B.

### 2.8. Statistical Analyses

The data were statistically analyzed via analysis of variance, followed by Bonferroni’s multiple *t*-test when applicable, using the Statcel4 software (OMS, Tokyo, Japan). Statistical significance was set at *p* < 0.05.

## 3. Results

### 3.1. Au_25_(SG)_18_ Suppresses PG Synthesis in Sparse Cultures of Vascular Endothelial Cells

**Figure 1:**
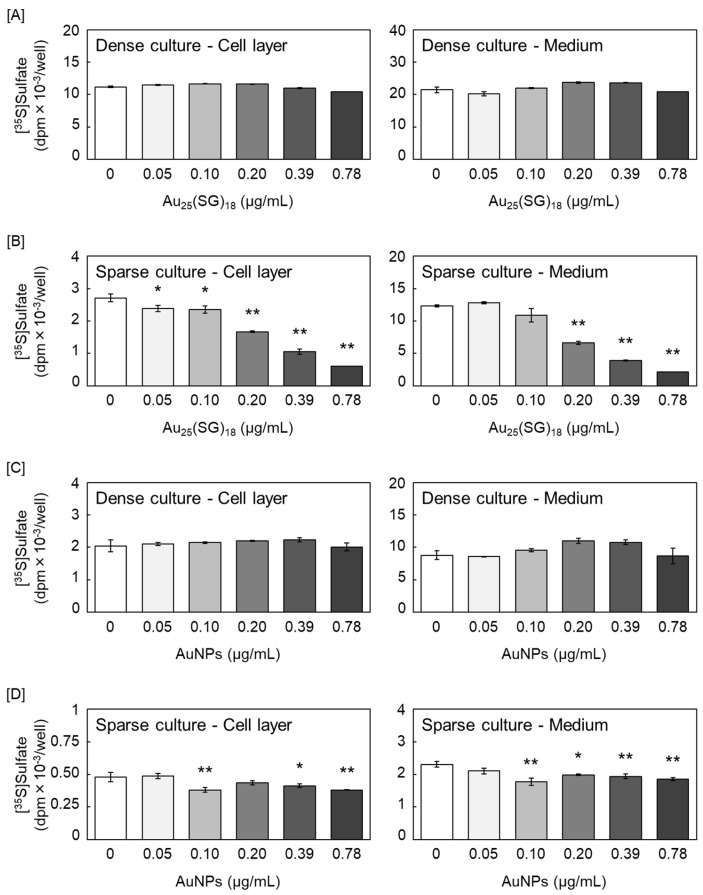
[^35^S]Sulfate incorporation into glycosaminoglycans (GAGs) accumulated in the cell layer (left panels) and conditioned medium (right panels). Bovine aortic endothelial cells in (**A**,**C**) dense and (**B**,**D**) sparse cultures treated with (**A**,**B**) Au_25_(SG)_18_ and (**A**,**D**) gold nanoparticles (AuNPs) at 0.05, 0.1, 0.2, 0.39, and 0.78 µg/mL for 24 h. Values are expressed as the mean ± standard error (S.E.) of four samples. * *p* < 0.05 and ** *p* < 0.01 vs. the corresponding control.

First, we investigated the effects of Au_25_(SG)_18_ and AuNPs on [^35^S]sulfate incorporation into the PGs synthesized by vascular endothelial cells. Au_25_(SG)_18_ did not modulate [^35^S]sulfate-labeled PG accumulation in the cell layer and conditioned medium of the dense cultures of vascular endothelial cells (Figure 1A). In contrast, in sparse cultures, Au_25_(SG)_18_ decreased [^35^S]sulfate-labeled PG accumulation in both the cell layer and conditioned medium in a concentration-dependent manner (Figure 1B). Notably, AuNPs did not affect PG synthesis in dense cultures (Figure 1C); however, they exerted similar but weaker effects than Au_25_(SG)_18_ in sparse cultures (Figure 1D).

### 3.2. Perlecan and Biglycan Expression Is Suppressed by Au_25_(SG)_18_ in Sparse Cultures of Vascular Endothelial Cells

**Figure 2:**
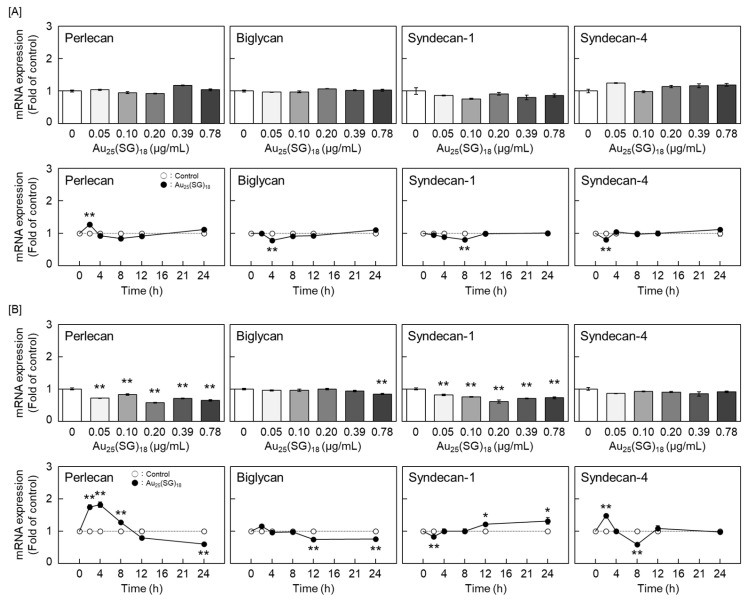
Perlecan, biglycan, syndecan-1, and syndecan-4 mRNA levels in vascular endothelial cells after treatment with Au_25_(SG)_18_. (**A**) Dense and (**B**) sparse cultures of bovine aortic endothelial cells incubated for 12 h in the absence or presence of Au_25_(SG)_18_ (0.05, 0.1, 0.2, 0.39, and 0.78 µg/mL; **upper panels**) or 2, 4, 8, 12, and 24 h in the absence or presence of Au_25_(SG)_18_ (0.2 µg/mL; **lower panels**). Values are expressed as the mean ± S.E. of four samples. * *p* < 0.05 and ** *p* < 0.01 vs. the corresponding control.

**Figure 3:**
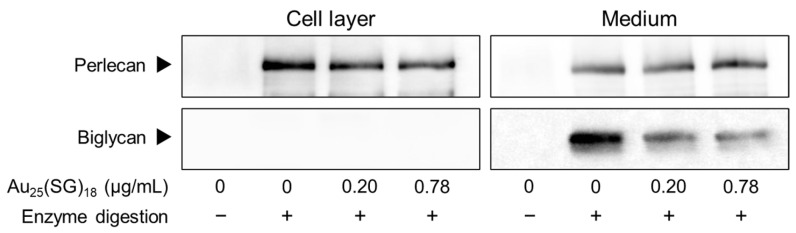
Perlecan and biglycan core protein levels in vascular endothelial cells after treatment with Au_25_(SG)_18_. Sparse cultures of bovine aortic endothelial cells were incubated for 24 h in the absence or presence of Au_25_(SG)_18_ (0.2 and 0.78 µg/mL).

Based on these results, we further explored the type(s) of PGs synthesized by vascular endothelial cells that were modulated by Au_25_(SG)_18_. In dense cultures, Au_25_(SG)_18_ showed no concentration- and time-dependent changes in any PG type (Figure 2A). In contrast, perlecan and biglycan mRNA expression levels were dose- and time-dependently decreased by Au_25_(SG)_18_ in sparse cultures (Figure 2B). Additionally, core protein expression levels of perlecan and biglycan were dose-dependently decreased by Au_25_(SG)_18_ in the cell layer and conditioned medium, respectively, in sparse cultures. Specifically, perlecan expression decreased to 76% and 69% of the control level at the 0.20 and 0.78 µg/mL of Au_25_(SG)_18_, respectively, and biglycan expression was reduced to 52% and 38% of the control level, respectively (Figure 3).

### 3.3. Au_25_(SG)_18_ Does Not Affect the mRNA Expression Levels of Perlecan and Biglycan in Dense Cultures of Vascular Endothelial Cells, Even at High Concentrations

**Figure 4:**
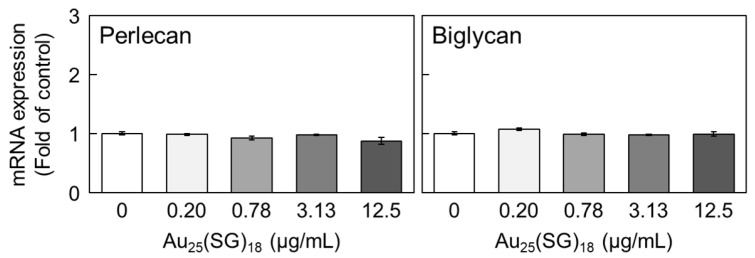
Perlecan and biglycan mRNA levels in dense cultures of vascular endothelial cells after treatment with Au_25_(SG)_18_. Dense cultures of bovine endothelial cells were incubated for 12 h in the absence or presence of Au_25_(SG)_18_ (0.2, 0.78, 3.13, and 12.5 µg/mL). Values are expressed as the mean ± S.E. of four samples.

We previously reported that Au_25_(SG)_18_ is less likely to accumulate in vascular endothelial cells at high cell density [51]. To determine whether this is why the expression levels of perlecan and biglycan were not reduced by Au_25_(SG)_18_ in our dense cultures, we examined the effects of high concentrations of Au_25_(SG)_18_ on the mRNA levels of these PGs. Notably, perlecan and biglycan levels were not affected by high concentrations of Au_25_(SG)_18_ (Figure 4).

### 3.4. Role of Arf6 in the Regulation of Cell Density-Dependent PG Synthesis

**Figure 5:**
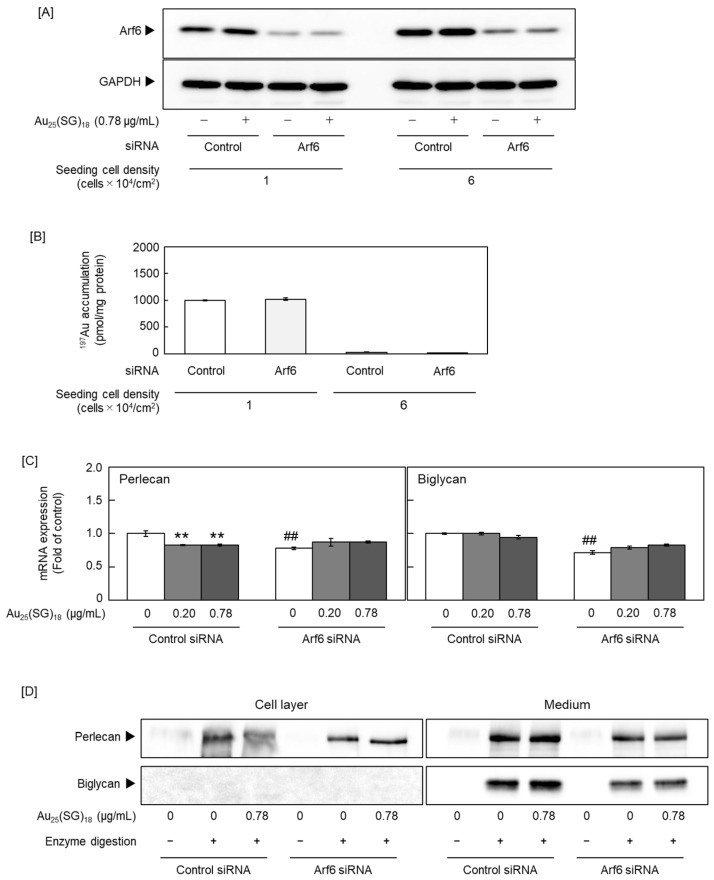
Effect of ADP-ribosylation factor 6 (Arf6) on the cell density-dependent suppression of proteoglycan synthesis by Au_25_(SG)_18_ in sparse vascular endothelial cells. (**A**) Arf6 protein expression and (**B**) intracellular accumulation of Au_25_(SG)_18_. Bovine aortic endothelial cells transfected with the negative control or Arf6 small interfering RNA (siRNA) were seeded at 1 and 6 × 10^4^ cells/cm^2^, cultured for 24 h, and treated with Au_25_(SG)_18_ (0.78 µg/mL each) for 24 h. Values are expressed as the mean ± S.E. of three samples. The (**C**) mRNA and (**D**) core protein levels of perlecan and biglycan. Bovine aortic endothelial cells transfected with the negative control or *Arf6* siRNA were seeded at 1 × 10^4^ cells/cm^2^, cultured for 24 h, and treated with Au_25_(SG)_18_ (0.2 and 0.78 µg/mL) for 24 h. Values are expressed as the mean ± S.E. of four samples. ** *p* < 0.01 vs. without Au_25_(SG)_18_; ^##^ *p* < 0.01 vs. Control siRNA.

**Figure 6:**
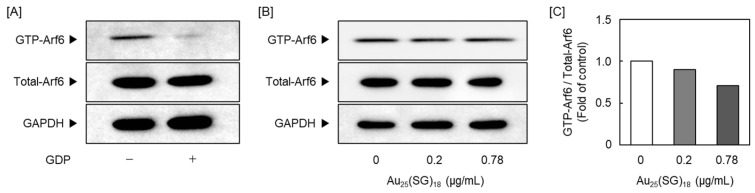
Arf6 activation in sparse cultures of vascular endothelial cells. Activated-Arf6 (GTP-Arf6) levels were measured via GGA3 pull-down and western blotting assays. (**A**) Confirmation of the experimental system. Sparse cultures of bovine aortic endothelial cells were harvested after incubation for 12 h. Negative control samples were reacted with GDP before pull-down assay. (**B**) Sparse cultures of bovine aortic endothelial cells were incubated for 12 h in the absence or presence of Au_25_(SG)_18_ (0.2 and 0.78 µg/mL). (**C**) Ratio of the intensity of GTP-Arf6 in (**B**) to that of Total-Arf6.

Because Au_25_(SG)_18_ regulated PG synthesis in a cell density-dependent manner, we focused on Arf6, a member of the Ras superfamily that includes small GTPases [52] and a GTP-binding multifunctional molecule activated at low cell density [53]. As Arf6 is involved in endocytosis, we examined whether Arf6 participates in the cell density-dependent cellular uptake of Au_25_(SG)_18_. We confirmed that Arf6 expression was suppressed by *Arf6* siRNA transfection with Arf6 protein levels reduced to approximately 25% and 35% of the control at seeding densities of 1 × 10^4^ and 6 × 10^4^ cells/cm^2^, respectively (Figure 5A) and compared intracellular gold accumulation between the Arf6-suppressed and control groups at low and high cell densities. Notably, no significant difference in gold accumulation was observed between the two groups, with no involvement of Arf6 in the cellular uptake of Au_25_(SG)_18_ (Figure 5B). Next, we examined whether Arf6 contributes to the suppression of perlecan and biglycan expression by Au_25_(SG)_18_ in sparse cultures. The mRNA levels of perlecan and biglycan were reduced by *Arf6* knockdown in the absence of Au_25_(SG)_18_. Perlecan suppression by Au_25_(SG)_18_ was maintained upon control siRNA transfection but abolished at the mRNA and core protein levels following *Arf6* knockdown. In contrast, the expression of biglycan mRNA and core protein was not suppressed upon siRNA transfection. The mechanism by which Arf6 affected biglycan expression could not be determined in this experimental system (Figure 5C,D). Specifically, in vascular endothelial cells treated with *Arf6* siRNA, no reduction in biglycan mRNA levels was observed following Au_25_(SG)_18_ treatment. Although the exact mechanism remains unclear, this phenomenon was confirmed in several experiments. In the steady state, Arf6 exists in an inactive form bound to GDP. When the cell receives stimuli such as growth factors or hormones, GDP dissociates from Arf6, and GTP binds to the site instead. Here, treatment with Au_25_(SG)_18_ suppressed GTP-bound Arf6 expression in endothelial cells in sparse cultures, without affecting total Arf6 expression (Figure 6). These results suggest that Au_25_(SG)_18_ reduces Arf6 activity in vascular endothelial cells at low cell density and suppresses perlecan expression by modulating downstream signaling.

## 4. Discussion

As the aorta consists of vascular endothelial cells, vascular smooth muscle cells and fibroblasts, the effects of chemicals on the aorta can be examined using these three cell types. However, vascular endothelial cells are the only cell type present in all types of blood vessels—macrovascular, microvascular, and capillary. Because blood vessels are ubiquitous in every organ and any chemical substance, regardless of its nature, must pass through the vascular endothelium before reaching its target organ, investigating the effects of chemicals on vascular endothelial cells may provide important insights into understanding the organ toxicity of these chemicals. Therefore, we investigated compound-induced endothelial toxicity and biological responses using vascular endothelial cells in our experimental model. Bovine aortic endothelial cells were used in this study because they are normal endothelial cells with properties similar to those of human endothelial cells, are easy to culture stably, and are well-characterized for proteoglycan synthesis. Endothelial PGs are biomolecules that play a crucial role in maintaining blood vessel homeostasis. Disruption of vascular homeostasis is closely associated with the initiation and progression of various diseases and conditions, such as cancer, stroke, and heart disease. Therefore, understanding PG synthesis mechanisms in endothelial cells, which regulate vascular functions, is important for vascular homeostasis. Although many studies have investigated the functions of PG species and their involvement in pathological conditions, only a few have focused on the mechanisms regulating their expression. In this study, we analyzed the regulatory mechanisms of PG synthesis in vascular endothelial cells using Au_25_(SG)_18_, which possesses no electrophilic properties. We found that: (1) Similar to AuNPs, Au_25_(SG)_18_ did not show cytotoxicity in endothelial cells, regardless of cell density. (2) Despite not being electrophilic, Au_25_(SG)_18_ accumulated in cells similarly to AuNPs, with higher uptake observed at low density. (3) Au_25_(SG)_18_ significantly suppressed perlecan and biglycan synthesis more potently than AuNPs. (4) Au_25_(SG)_18_ suppressed perlecan and biglycan expression only in endothelial cells at low cell density. (5) Au_25_(SG)_18_ suppressed perlecan expression via Arf6. (6) Arf6 was involved in the induction of perlecan expression in endothelial cells at low cell density. Collectively, these results suggest that Au_25_(SG)_18_ negatively modulates perlecan and biglycan synthesis in vascular endothelial cells at low cell density, without causing cytotoxicity. Notably, Au_25_(SG)_18_, which is not an electrophilic substance, accumulated and affected PG synthesis in vascular endothelial cells at low cell density. Furthermore, these effects were not observed with AuNPs, suggesting that the negative modulation of perlecan and biglycan synthesis in vascular endothelial cells is unique to Au_25_(SG)_18_. However, since Arf6 expression is not specific to vascular endothelial cells [54], if Au_25_(SG)_18_ is taken up under similar conditions, the expression of proteoglycan species other than perlecan and biglycan in other cells may be conceivably regulated.

Gold compounds, such as gold thiolates [55] and auranofin [56], are widely used in medicine to treat rheumatoid arthritis. Recently, their applications have been extended to other areas such as biological diagnostics using nanoscale gold particles [57], drug delivery [58], bioimaging [59,60], and radiation therapy [61,62]. With the accelerated development of gold-containing NPs, research on their toxicity and in vivo distribution has also increased. Accumulation of AuNPs in cells is affected by their size, charge, and surface modifications; AuNPs larger than 50 nm cannot pass through the reticuloendothelial system barrier [63,64], whereas those smaller than 50 nm accumulate in cells [65]. Positively charged AuNPs are highly toxic [66] and induce cell death [67]. Notably, AuNP charge can be modified by their coating, with neutral AuNPs exhibiting lower cellular uptake than negatively charged AuNPs [68,69]. These results suggest that the size and charge of NPs are important for their biological activities, including cytotoxicity upon intracellular accumulation. Au_25_(SG)_18_ exhibits a diameter of approximately 1 nm [47] and low intramolecular polarity; therefore, it possibly accumulates easily in cells and exhibits low cytotoxicity. A previous study reported low cytotoxicity of Au_25_(SG)_18_ in vascular endothelial cells [51]; in this study, its intracellular accumulation was lower than that of AuNPs because of its lack of charge. Nonetheless, it markedly suppressed perlecan and biglycan expression in vascular endothelial cells in sparse cultures. These results suggest that Au_25_(SG)_18_ exhibits higher biological activity than AuNPs. Although the mechanisms underlying the biological activities of Au_25_(SG)_18_ remain unknown, it is a useful analytical tool to elucidate the regulatory mechanisms of PG synthesis in vascular endothelial cells.

Previous studies have reported that the internalization of lipoic acid-protected gold nanoclusters into cells occurs via endocytosis, which is involved in the nutrient uptake and metabolic turnover of cell surface molecules [70,71]. Clathrin and caveolae are molecules that regulate the formation of endocytic vesicles [72,73]. Based on the morphological characteristics, endocytosis is broadly classified into four types: (i) Clathrin-dependent endocytosis, (ii) caveolae-dependent endocytosis, (iii) clathrin/caveolae-independent endocytosis, and (iv) macropinocytosis, which is associated with the dynamics of the actin cytoskeleton [74]. Clathrin-mediated endocytosis is not affected during mitosis, and clathrin internalized during this process does not return to the membrane surface until late telophase [75]. In contrast, caveolae are membrane structures commonly found in endothelial cells [76]. Caveolin, a key molecule forming caveolae, actively translocates into cells during cell division and localizes to endosomal structures. It subsequently returns to the cell surface during or after cytokinesis [77] and regulates nutrient and receptor transport in proliferating cells [78]. These reports suggest that clathrin-mediated endocytosis is unaffected by the cell cycle, whereas caveolae-mediated endocytosis is more likely to occur during mitosis in endothelial cells. Therefore, Au_25_(SG)_18_ translocates into cells via caveolae-dependent endocytosis, which is negatively dependent on the cell density.

Synthesis of PGs, particularly syndecans and transmembrane-type small HSPGs, influences membrane trafficking and recycling [79,80]. Whether Arf6 is involved in the synthesis of perlecan, a large HSPG that exists in the extracellular matrix of vascular endothelial cells, remains unclear. However, Arf6 possibly regulates perlecan synthesis, as Arf6 has been reported to regulate the synthesis of fusogenic lipids, which are involved in exocytosis, a process related to perlecan secretion [79]. In this study, Au_25_(SG)_18_ significantly suppressed perlecan synthesis in vascular endothelial cells, indicating that Arf6 plays a key role in the regulation of perlecan secretion.

Arf6 is activated by converting its bound GDP to GTP, and this exchange is triggered by guanine nucleotide exchange factors (GEFs) [81]. Arf6 returns to its inactivated state by hydrolyzing bound GTP to GDP through its own GTPase activity. Typically, GTPase activity of small G proteins, including Arf6, is very low; therefore, assistance from a GTPase-activating protein is essential for the hydrolysis of GTP bound to Arf6 into GDP [82]. Therefore, both GEFs and GTPase-activating protein are important for maintaining Arf6 function [83]. In this study, perlecan suppression by Au_25_(SG)_18_ was abolished in vascular endothelial cells, further suppressing Arf6 expression. Additionally, Au_25_(SG)_18_ reduced the amount of GTP-bound Arf6, suggesting that Au_25_(SG)_18_ negatively regulates perlecan synthesis by inhibiting the activity of Arf6 GEFs, such as the cytohesin family members [84].

We previously reported that heavy metals and organic–inorganic hybrid molecules regulate PG expression via MAPKs, which regulate cell proliferation, differentiation, and growth. Lead suppresses perlecan expression via the epidermal growth factor receptor–ERK1/2–cyclooxygenase-2–prostaglandin I_2_ pathway [29] and copper complex copper(II) bis(diethyldithiocarbamate)-induced syndecan-4 expression mediated by p38 MAPK [31]. However, among the representative MAPKs (ERK1/2, JNK, and p38 MAPK), Au_25_(SG)_18_ suppressed the activation of ERK1/2 but had no effect on the activation of JNK and p38 MAPK (Figure S2). This suggests that Au_25_(SG)_18_ suppresses perlecan and biglycan expression independently of the MAPK pathway. Perlecan is a component of the basement membrane [9] and promotes the binding of FGF-2 to receptors on endothelial cells via heparan sulfate chains, thereby enhancing their proliferation and migration [85]. Arf6 stimulates epithelial cell migration [86], and its activation promotes fibroblast proliferation [87]. Although the detailed regulatory mechanisms remain unclear, these findings suggest that Arf6 regulates the proliferation and migration of endothelial cells, contributing to the maintenance of vascular homeostasis. Overall, this is the first study to reveal that Au_25_(SG)_18_ regulates perlecan expression, identifying Arf6 as an important factor in the regulation of vascular endothelial cells, thereby highlighting non-electrophilic organometallic compounds as useful tools for biological function analysis.

## 5. Conclusions

This study demonstrated that Au_25_(SG)_18_, a gold cluster with low electrophilicity, suppresses proteoglycan expression in vascular endothelial cells under low-density culture conditions. This cluster specifically downregulates perlecan by inactivating Arf6, which indicates that Arf6-mediated extracellular transport is a key regulatory mechanism. Although biglycan expression is reduced, the underlying mechanism remains to be elucidated. Collectively, these findings highlight Arf6 as an important pathway in endothelial proteoglycan synthesis.

## Data Availability

The data supporting of this study are included in the article. Further inquiries can be directed to the corresponding authors.

## Supplementary Materials

The following supporting information can be downloaded at: https://www.mdpi.com/article/10.3390/cells15020209/s1, Figure S1: An overview of the entire experimental workflow. Figure S2. Activation of MAPK proteins in vascular endothelial cells after treatment with Au_25_(SG)_18_.

## Abbreviations

The following abbreviations are used in this manuscript:

Arf6	ADP-ribosylation factor 6
BCA	Bicinchoninic acid
CSPG	Chondroitin sulfate proteoglycan
DMEM	Dulbecco’s modified Eagle’s medium
DSPG	Dermatan sulfate proteoglycan
ERK	Extracellular signal-regulated kinase
FGF	Fibroblast growth factor
GAG	Glycosaminoglycan
GEF	Guanine nucleotide exchange factor
HSPG	Heparan sulfate proteoglycan
MAPK	Mitogen-activated protein kinase
PG	Proteoglycan
TGF	Transforming growth factor
