# Supplementary material for: Cell Density-Dependent Suppression of Perlecan and Biglycan Expression by Gold Nanocluster in Vascular Endothelial Cells
Source: Cells. 2026 Jan 22;15(2):209. doi: 10.3390/cells15020209 (PMC12840218; doi:10.3390/cells15020209)
Supplement: Supplementary file 1 [file cells-15-00209-s001.zip › cells-4074139-supplementary.pdf]

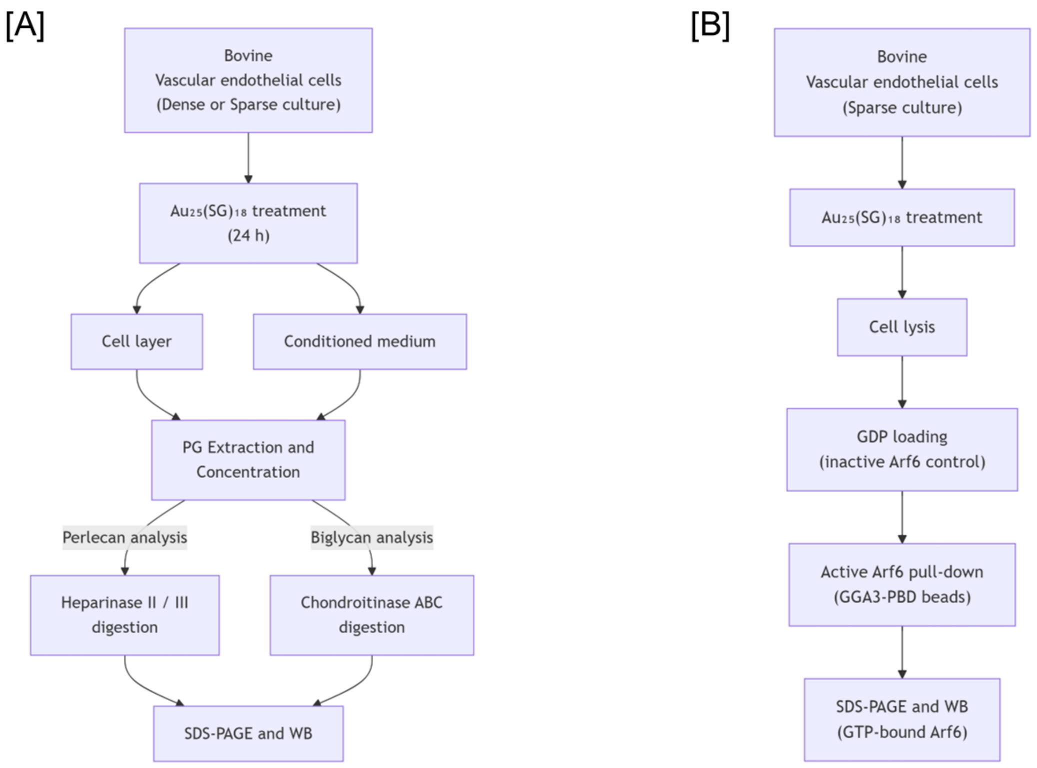

Figure S1. An overview of the entire experimental workflow. [A] PG core protein expression and western blotting analysis (Section 2.5) and [B] Arf6 activation assay (GTP-bound Arf6 pull-down; Section 2.7).

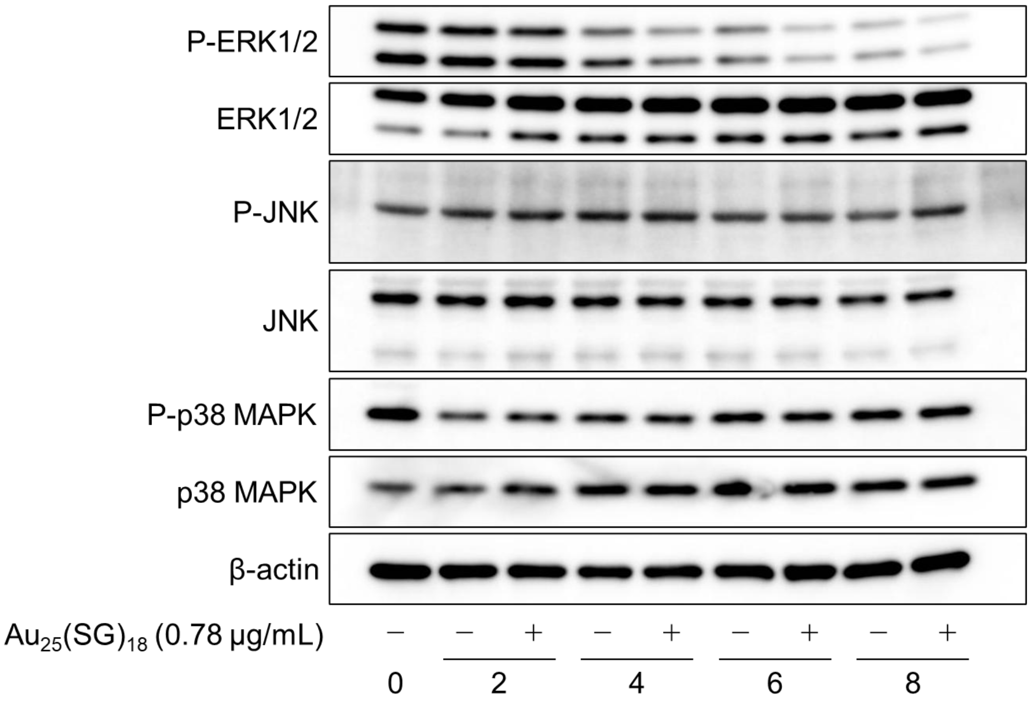

Figure S2. Activation of MAPK proteins in vascular endothelial cells after treatment with $Au_{25}(SG)_{18}$. Sparse cultures of bovine aortic endothelial cells were incubated for 24 h in the absence or presence of $Au_{25}(SG)_{18}$ (0.78 μg/mL).
